# What are the economic dimensions of occupational health and how should they be measured? A qualitative study

**DOI:** 10.1186/s12889-022-13659-y

**Published:** 2022-07-15

**Authors:** Nathanael Lutz, Lena Dalle Grave, Dirk Richter, Tom Deliens, Nick Verhaeghe, Jan Taeymans, Peter Clarys

**Affiliations:** 1 Department of Movement and Sport Sciences, Vrije Universiteit Brussel, Brussels, Belgium; 2 Department of Health Professions, Bern University of Applied Sciences, Bern, Switzerland; 3 Center for Psychiatric Rehabilitation, Bern University Hospital for Mental Health, Bern, Switzerland; 4 Department of Public Health and Primary Care, Interuniversity Centre for Health Economics Research (I-CHER), Ghent University, Ghent, Belgium; 5 Research Institute for Work and Society - HIVA, KU Leuven, Leuven, Belgium

**Keywords:** Occupational health, Cost-Benefit analysis, Costs and cost analysis, Indirect costs, Organizational efficiency, Intangible benefits

## Abstract

**Background:**

Decision makers want to know if there is a financial benefit in investing scarce resources in occupational health management (OHM). Economic evaluations (EEs) of OHM-strategies try to answer this question. However, EEs of OHM-strategies which are strongly marked by quantitative methods may be limited by contextual, qualitative residuals. Therefore, the objectives of this study were to (1) explore important economic dimensions of OHM and (2) to discuss the methods used in current EEs for measuring these dimensions.

**Methods:**

In this explorative qualitative study, OHM-specialists were recruited via the Swiss organisation for health promotion. Thirteen semi-structured interviews were performed from November 2020 until May 2021. Videotapes were transcribed verbatim and organised by using an open coding strategy. Codes were clustered and synthesised as themes (i.e. the dimensions of EEs of OHM) through a mix of inductive and deductive content analysis. Member check with eight participants was accomplished to validate the results.

**Results:**

The interviews had an average duration of 70.5 min and yielded 609 individual codes. These codes were merged into 28 subcategories which were finally categorised into five main themes: Understanding of OHM, costs, benefits, environmental aspects, and evaluation of OHM. Participants stated that the greater part of costs and benefits cannot be quantified or monetised and thus, considered in quantitative EEs. For example, they see a culture of health as key component for a successful OHM-strategy. However, the costs to establish such a culture as well as its benefits are hard to quantify. Participants were highly critical of the use of absenteeism as a linear measure of productivity. Furthermore, they explained that single, rare events, such as a change in leadership, can have significant impact on employee health. However, such external influence factors are difficult to control.

**Conclusions:**

Participants perceived costs and benefits of OHM significantly different than how they are represented in current EEs. According to the OHM-specialists, most benefits cannot be quantified and thus, monetised. These intangible benefits as well as critical influencing factors during the process should be assessed qualitatively and considered in EEs when using them as a legitimation basis vis-à-vis decision makers.

**Supplementary Information:**

The online version contains supplementary material available at 10.1186/s12889-022-13659-y.

## Background

Adults spend most of their waking time at work which significantly influences their life and their health. However, establishing the causal relationship between work and health is not straightforward, it is complex and bidirectional [1]. On the one hand, high job-demands and low job-resources are associated with deteriorated health [2]. For example, a systematic analysis from 195 countries found that over 700,000 deaths and more than 23 million disability-adjusted life years from ischemic heart disease and stroke combined were attributable to long working hours [3]. On the other hand, longitudinal population studies and meta-analyses showed that poor health influences the quality and quantity of work [4, 5]. For example, a study across the UK, France, Spain, Germany and Italy found that severe daily pain is related to a 20-point reduction in the probability of being full time employed [6]. This has important economic consequences. In the European Union, work-related injuries and illnesses result in the loss of 3.3% of its gross domestic products (GDP) which corresponds to €476 billion yearly [7].

Occupational health has its roots in the British Health and Safety at Work Act (1974), which represents the basis of health and safety legislation in many countries today. The first interventions primarily addressed safety hazards such as heat and cold stress or environmental toxins (i.e. occupational safety). From the 1980s onwards, health promotion and disease prevention became increasingly popular, typically focusing on worksite nutrition, exercise, and smoking (i.e. occupational health promotion (OHP)). Despite the implementation of many OHP interventions, its sustainability is still disputed because they typically depend on other factors such as leadership support or work organisation (i.e. occupational health management (OHM)) [8].

The most common barrier for decision makers to invest in OHM is budget constraints. To assess if the benefits of OHM justify its costs, economic evaluations (EEs) of OHM-strategies gained importance over the last two decades [9–14]. Reviews identified two main types of EEs in the field of OHM: Cost-benefit analyses (CBAs) and cost-effectiveness analyses (CEAs). CBAs measure costs and effects in monetary terms, allowing to report a return-on-investment (ROI) of an intervention. For example, costs of a stress management program among employees were reported to be €299 per person while the benefits due to increased productivity were €488 per person. This yielded a net monetary benefit of €189 per employee, corresponding to an ROI of 63.3% [15]. CEAs compare the incremental costs and the incremental effects of at least two alternatives (e.g. OHM-program versus no OHM-program). CEAs typically calculate an incremental cost-effectiveness ratio (ICER) which represents the additional costs per additional unit of effect. For example, van Wier et al. [16] conducted a CEA of a weight control program among employees. The costs were €352 higher in the intervention group as compared to the control group, but on average, employees in the intervention group lost 0.29 kg more. This resulted in an ICER of €1009 per extra kilogram weight lost. The decision if such a weight control program is cost-effective depends on the willingness-to-pay (i.e. how much money the employer is willing to pay for one extra kilogram weight reduction of an employee).

Overall, the findings from reviews regarding EEs of OHM are inconsistent. Furthermore, a number of reviews identified a negative relationship between methodological quality of the study and ROI [9, 11, 13, 17]. Typical conclusions of these reviews are that studies were informed by a narrow perspective, e.g. only the costs for the employer were considered but the costs for society were omitted. For example, medical costs are not relevant from the employer’s perspective, while they become important from a societal perspective. Thus, taking multiple perspectives into account is important because in certain situations it can legitimise an intervention being cross-subsidised, e.g. by the public sector, because otherwise it would not be implemented. Further it was criticised that time horizons have been chosen inappropriately and that the benefits of OHM (mostly reduced to sick leave) were defined and analysed in different ways. In turn, these reviews call for a more comprehensive investigation and reporting of the economic outcomes of OHM to identify aspects of OHM that reach well beyond intervention costs and sick leave rates.

To the best of our knowledge, there is no study that investigated the economic dimension that should be included in EEs of OHM interventions. Following a participatory approach, we therefore aimed to examine the views and opinions of OHM stakeholders regarding the economic dimensions of OHM. The current inquiry addressed knowledge and appraisals of OHM-specialists in Switzerland with the aims to:

1. identify and to put into context important economic costs or benefits of OHM that would be omitted or not provided with priority in current EE frameworks.
2. discuss the methods used for measuring these costs and benefits.

A qualitative approach was chosen because EEs of OHM-interventions, strongly marked by quantitative methods, may be limited by contextual, qualitative residuals [18]. Research underlines the importance of qualitative approaches in the field of EEs to gain detailed knowledge about complex processes and contexts. Coast [19] emphasised the explicit application of qualitative methods in health EEs to determine all relevant costs and benefits (i.e. dimensions).

## Methods

The methods were reported following the Consolidated criteria for reporting qualitative research (COREQ) [20].

### Participants

To achieve our objectives, we discussed the common methods of EEs in the field of OHM with OHM-specialists from different companies. Interviewees were recruited with support from Health Promotion Switzerland (HPS), a national foundation which has the governmental mandate to stimulate, coordinate, and evaluate health promotion programs [21]. OHM is one of the core areas of HPS. HPS offers companies various instruments, services, and trainings to implement and evaluate comprehensive OHM. Companies who systematically apply OHM and fulfil the criteria set by HPS, receive the award “Friendly Work Space” (FWS) [22]. Each company with the FWS label has at least one OHM-specialist who is responsible for implementing and evaluating OHM strategies. These OHM-specialists were contacted by e-mail via HPS and asked to participate in an online interview. Additionally, participants were requested to suggest potentially eligible interview candidates (i.e. snowball sampling), which were then contacted by the interviewer (first author of this manuscript). The inclusion criteria were: the OHM-specialist (1) is/was responsible for implementing and/or evaluating OHM-activities in a company, (2) has at least two years of experience in the field of OHM and (3) is familiar with the evaluation concept provided by HPS (common ground). To ensure sufficient diversity of opinion, companies from different work sectors, and different sizes were contacted. There were no restrictions regarding company size, company sector or professional background of the participants.

One third of the invited persons responded positively. Time restrictions was the only reason for refusing to participate. Ten interested persons did not fulfil the inclusion criteria and were not interviewed. Table 1 provides descriptive statistics of participants and their companies.

**Table 1:** Characteristics of interview participants

Companies (*n* = 13)	Field of activity (n)	
	◦ Insurance	4
	◦ Public transport	2
	◦ Science	1
	◦ Food processing	1
	◦ Education	1
	◦ OHM consulting^a^	4
	Employees (median, min, max)	2500, 56, 35’000
	Public Sector (n)	3
Participant characteristics (*n* = 13)	Age in years (median, min, max)	49, 28, 61
	Experience in OHM in years (median, min, max)	12, 2, 20
	Gender (n females)	7
	Background (n^b^)	
	◦ Psychology	4
	◦ Health promotion	4
	◦ Human resources	3
	◦ Sociology	3
	◦ Law	2
	◦ Economy	2
	◦ Quality management	1
	◦ Social insurance	1
	Academic degree (n)	
	◦ PhD	2
	◦ Master	9
	◦ Other	2

^a^Four participants worked in various companies as OHM-specialists during their career and worked as consultants in the field of OHM at the time of the study

^b^Multiple responses per person

### Data collection

A total of 13 semi-structured face-to-face interviews with open-ended questions were performed between November 2020 and May 2021. Each person was interviewed once. Due to the COVID-19 pandemic, interviews were held and recorded using MS Teams®. To ensure not to miss any ‘new’ information, one additional interview was conducted after theoretical saturation was reached. All participants provided informed consent prior to the interview and the local ethics committee approved the study. The semi-structured interview-guide (see Additional file 1) was developed following the five-step framework including (i) identifying the requirements for using this type of interviews; (ii) identifying and applying previous knowledge; (iii) preparing the provisional semi-structured interview guide, (iv) testing the guide, and (v) presenting the final semi-structured interview guide [23]. The study aims and methods were discussed with one OHM-specialist prior to the development of the actual interview guide. This ensured that aim, methods, and interview-questions were goal-oriented and well-coordinated. The first interview served as a field-test of the interview-guide to confirm the coverage and relevance of the content of the formulated questions as well as the anticipated timing (about 60 min) [24]. All interviews were led by a moderator (first author), a male researcher in the field of economic evaluations of occupational health interventions. The moderator did not know any of the participants prior to the interviews. In a short introduction, the moderator introduced himself, summarised the main findings from previous studies in this field, indicated the knowledge gap, and explained the goal of the study.

### Analytical approach, data analyses quality assurance

We performed a content analysis to establish themes (i.e. the economic dimensions of OHM). A mix of an inductive and deductive approach was applied. The inductive part is justified by the fact that no pre-determined coding-frame was applied, and that part of the interview guide consisted of open-ended questions, which were not motivated by previous expertise from the literature. However, some questions in the interview guide resulted from knowledge and assumptions of the authors, based on previous research activities in this field (e.g. [11, 17]). Furthermore, the nature of the topic is cause-and-effect oriented. Therefore, a deductive approach was dominant.

Data obtained from the videotapes were transcribed verbatim and synchronised with the video files. This allowed consideration of non-verbal reactions of participants. Transcribing is an important step of analysing the data [25], and was therefore performed by the first author.

After repeated reading of the transcripts, all quotes were represented by codes. To maximise reliability of data interpretation, analyses were carried out by two researchers (first and second author) independently. The second author is a female researcher who is familiar with qualitative methods and who has a background in occupational health management, economics, and social sciences. First, all codes were discussed and adjusted if appropriate. After systematically allocating all codes into clusters, appropriate labels were determined for each cluster. The clusters were synthesised as appropriate and then organised into levels, with the first level representing the final themes [26]. Doubts or disagreements were discussed with members of the research team until consensus was reached. The final themes were discussed with eight participants in order to validate the findings (i.e. member check) [27]. Transcript analysis was conducted within one week after the interview to decide on saturation of information. NVivo (Version 12.5) software was used for the analysis of the transcripts.

## Results

Theoretical saturation was reached after 12 interviews, indicated by the fact that no new codes related to a higher-level theme were established [28]. One additional interview was conducted to ensure that saturation was reached. The average duration of the interviews was 70.5 (sd = 14.8) minutes. Transcripts and reports yielded a total of 609 individual codes and 1537 references. In the final coding framework, these codes were allocated into 28 subcategories, which were synthesised into five main themes: Understanding of OHM, costs of OHM, benefits of OHM, environmental aspects of OHM, and evaluation of OHM (Table 2).

**Table 2:** Overview of codes, subcategories and the five final themes

Main themes	Subcategory 1	Subcategory 2	Subcategory 3	Total number of codes	Total number of references
Understanding of OHM	What is OHM (21)			21	50
Costs of OHM	Direct costs (11)			205	525
	Indirect costs (201)	Attitudes towards OHM (18)			
		Culture (100)	Culture-inhibiting (36)		
			Culture-promoting (64)		
		Convincing leadership (14)			
		Integration into existing structures (30)			
		Other costs (20)			
Benefits of OHM	Corporate success (89)	Qualitative outcomes (43)		125	328
		Quantitative outcomes (46)			
	Resources and demands (17)	Health (18)			
Environmental aspects of OHM	Company’s environment (9)			10	33
	Employees’ environment (8)				
Evaluation of OHM	Arguments against economic evaluations (25)			264	663
	Chances of economic evaluations (25)				
	Methodological aspects (180)	Data collection (29)	Quantification of benefits (33)		
		Study design (34)			
		Perspectives (9)			
		Modelling approaches (22)			
		Impact models (73)			
		Time horizon (7)			
		Target population (6)			

The number in brackets indicate the number of codes for each subcategory. The codes do not sum up to total codes because some codes were allocated into more than one subcategory

**Fig. 1:**
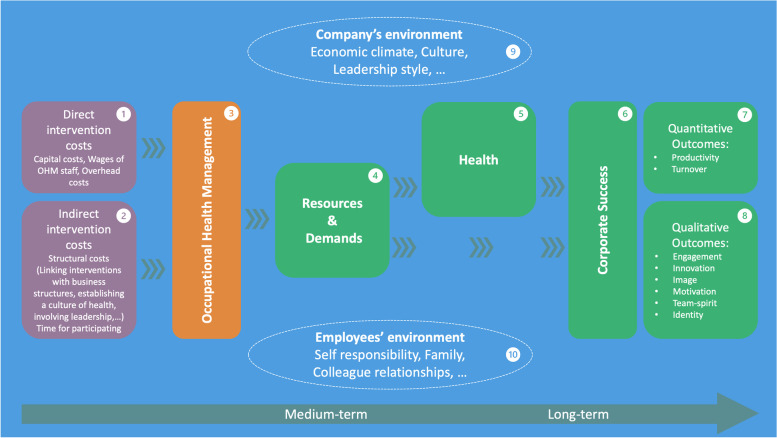
Impact-model for economic evaluations of occupational health management, based on the organisational health development research model [29]

The five main themes, as well as other key findings from the interviews, are illustrated in Fig. 1.

The presented model is based on the organisational health development research model [29], but was adapted for EEs of OHM according to the findings from the interviews. The colours indicate the main themes which were identified through the interviews: understanding by OHM (orange), costs of OHM (purple), the benefits of OHM (green) and environmental factors (blue). Each component of OHM, including the processes (arrows), is affected by environmental factors (9 & 10). According to the participants and as illustrated in this model, economic evaluations that only focus on direct costs (1) and monetisable outcomes (7) are limited. With regard to comprehensive economic evaluations, participants strongly advise to also consider indirect costs (2), qualitative outcomes (8), as well as critical events during the process (i.e. leadership change). Moreover, health (5) and corporate success (6) can only be influenced in the long term (several years). However, participants believe that the assessment of resources and demands (4) in the medium term may be sufficient as they see the ratio of resources and demands as ground-breaking for health and corporate success. In the following description of the results, reference is made repeatedly to Fig. 1

### Understanding of occupational health management

According to the participants, many people have a wrong idea of OHM and confuse OHM with “traditional” occupational safety and OHP: “*[…] when people hear of OHM they think of free apples and lunch hour yoga (P11)*”. Participants strongly doubt that isolated OHP interventions work without them being conceptually linked with the structures of the organisation: “*I know a lot of people who do OHM and only focus on health promotion. And you must be aware that this is something completely different. Health promotion is not bad per se, but it’s like a drop in the bucket (P1)*”. For this reason, OHP is an essential, but not a sufficient component for comprehensive OHM (Fig. 1, marking 3). Regarding EEs, this means that the “management-part” must be taken into account. However, it is difficult to draw a clear line between what belongs to OHM and what does not: *“[…] leadership training has an extreme impact on employees’ health but is not considered OHM for us (P11)”.* This is relevant for EEs because many measures are not considered OHM but contribute to health: *“In the end, everything somehow affects health”.*

### Costs of OHM

#### Costs of interventions (direct costs)

Participants understand some of the costs as direct costs: *“There are relatively evident costs like a consultant; you have to pay for or an online tool that you buy or something like that. The direct costs, yes, they are evident (P3)”* (Fig. 1, marking 1). Such capital costs (e.g. wages for OHM personnel, offers purchased externally) or overhead costs (e.g. electricity or cleaning) are covered by a fixed budget that is available for OHM in most companies. Again, a critical comment in this regard is that it is not clear what is counted as OHM: *“[…] then, the question really is where to start counting. For example, we have a day care centre […]. Or I could say that we have an excellent restaurant. […] We have a company ambulance, the whole emergency organisation, this is huge! (P10)”.*

#### Establish and maintain a culture of health (indirect costs)

*“Healthy employees as the key to success (P2)”* must be the basic attitude for a company in order for OHM to function sustainably: *“I simply believe that more should happen by conviction and from the mindset (of the management) than the naked belief in numbers (P1)”*. This attitude is the basis for a “culture of health” in which health is discussed and social dynamics are created: *“If you participate, then it works. […]. The problem is that nobody does. And this only happens when social dynamics are created (P3)”.*

To attain this, one must “*convince every day anew about the importance (P2)”* and the culture of health must be “*constantly lived and cultivated (P1)*”. In this context, leadership is described by the participants as the biggest lever in OHM: *“I think there lies a lot (of potential) whether OHM has a benefit or not. From my chair, I can only make a little difference. It is the lead person who has it in his hands to deal with the employee in such a way that he remains healthy and performs at his best (P13)”.* All these efforts for creating and maintaining such a culture were referred to as indirect, invisible, or structural costs for the organisation (Fig. 1, marking 2). Indirect costs for the employees themselves (time for participating in OHM interventions, motivation, personal responsibility) were also reported. As illustrated in Fig. 1, participants rate the indirect costs higher than the direct costs. However, an additive approach to indirect costs does not seem to be an adequate method for EEs: *“Whether these indirect costs should now be included to the same extent is a matter of debate. So, you can do the cost-benefit calculation with these costs and can also do it without. And I tend to say that this should be viewed with caution. So, this is my stance (P3)”.*

### Benefits of OHM

#### Job-demands, job-resources, and health

From the participants’ perspective, OHM-interventions should reduce job-demands and promote job-resources of employees (Fig. 1, marking 4). Demands and resources are considered as starting point for health and other outcomes of OHM: *“We only show the resource-demands-ratio. Resources, demands, stress, health. These interdependencies are there. So, we don’t have to show that people are healthy or healthier […] (P3)”* (Fig. 1, marking 5). Responsibilities are another important issue: *“We have discussed so many times: who is responsible for health? Who should pay in the end? I don’t know either. (P6)”.* From the participants’ perspective, the employer should take responsibility for job-demands and job-resources: “*[…] to influence health and motivation. That is to understand quite well what the demands in a system are and what the resources (P2)”.*

#### Corporate success

Regarding business success, the participants distinguish between soft (i.e. intangible) and hard outcomes. As illustrated in Fig. 1 (marking 5), the OHM-specialists defined productivity (reduced absenteeism and presenteeism) and involuntary turnover as hard outcomes (i.e. quantitative outcomes). Absenteeism represents an important metric in the interviews: *“The reason why we often take absences as an outcome is because you can put it very nicely in an excel spreadsheet (P5)”.* However, there is great scepticism whether absenteeism should be used linearly as a measure of productivity: “*One day of absence may be absolutely unproblematic for a company. Maybe even positive, if someone skips a Monday, then he returns even more well rested to work (P3)*”. In a similar manner, presence at work does not automatically mean productive time (i.e. presenteeism). *“[…] I can work an hour while being highly productive […]. And I can work one hour and check my Facebook messages. So, one hour is not one hour (P4)”.* Benefits such as engagement, innovation or team-spirit were perceived as intangible outcomes (Fig. 1, marking 8): “[…] *don’t capture any pseudo-accurate numbers… It’s just not all monetisable or quantifiable (P1)”.* Thus, for example, it is difficult to consider intangible outcomes in EEs: *“You know, there is also this ROI. That’s good and well, but what concrete indicators do you hang it on? (P10)”.*

Participants believe that the greater part of the company-benefits is not measurable: *“[…] then you have to really think carefully what you quantify and what, and this is the larger part of the effect, what you don’t quantify* (P1)”. Moreover, they believe that intangible outcomes (e.g. lack of motivation of the employees) have a stronger impact on the company’s success than hard outcomes (e.g. absences): *“A company goes bust because of the invisible costs, not because of the visible costs (P11)”*.

### Environmental aspects of OHM

Environmental factors of the company (Fig. 1, marking 9), as well as those of the employees (Fig. 1, marking 10), can strongly influence the effectiveness of OHM and its evaluation: *“You can interpret it as you like. Is it coincidence? Is it a restructuring? There are so many reasons… a bad summer… There are just so many things where you can never say concretely, that’s why (P7)”.* The participants describe the constant, increasingly rapid changes in the environment as a great challenge: “*Where it should go with the future working world. I think that’s the same for everyone. How will we work in the future? Yes, I don’t know (P3)”.* The COVID-19 pandemic is a good example that often measures must be implemented without having time to develop them adequately: “*Simply because the world is developing so fast. Often you don’t even start with a prototype anymore but with this minimum viable product (P8)”.* Thus, environmental factors may impact every single component of OHM.

### Evaluation of OHM

#### Arguments against economic evaluations

For the participants it is clear that *“healthy employees can only be good for a company (P8)”.* This statement can be interpreted in a way that OHM must absolutely be economical in the long-term, at least from a societal perspective. It turns out that especially among more experienced participants, this conviction of OHM is more pronounced than among less experienced ones. The value of meaningfulness, conviction, and culture is rated decidedly higher than facts based on EEs: *“It’s a cultural thing. That’s something that is important to us. We don’t want to monetise that at all. As soon as we bring it to an accountant’s logic, it loses value (P4)”*. “*You don’t have to evaluate everything, certain things we just do (P5)”* represents a typical statement. They believe that EEs can never represent the true costs and benefits: “*You just don’t have to feel that it’s the plain truth […]. It’s not, […] you can’t just count the visible costs (P10)”.* A pure focus on monetary benefits can even be counterproductive: “*Organisations, that primarily ask for the return-on-investment, have quite bad prospects for a successful (OHM) strategy (P1)”*. 

### Chances of economic evaluations

Participants see the opportunity of EEs exclusively in having a basis of legitimacy toward decision makers: “*It would offer a great deal of legitimacy if we could argue with economic facts and figures, which we cannot do (P9)”.*

In some companies, cost-analysis was used to raise awareness among decision makers and to estimate how much to invest in specific interventions: “*This is our average wage, that is the personnel costs. And there is a study where it says, 1.5 to 5 times the direct wage costs are the indirect costs of someone who is absent. […] and then that easily adds up to one million. An employee who drops out for two years, direct and indirect costs, one million! And then I said: dear friends, at this moment we have 20 people in our reintegration program. That is 20 million (P10)”.*

####  Methodological aspects 

##### Data collection

Surveys among employees are by far the most common method of collecting data. This data is needed for diagnostics, action planning, and evaluation. The participants describe surveys as time consuming and thus, expensive, and often the response rate is unsatisfactory. Surveys must appear genuine, and the employees must feel that something is really happening on the basis of their feedback (i.e. evaluation culture): “*People really have to have the feeling that they are being questioned and that something will happen. And this is often the problem, […] then nothing happens. And of course, that doesn’t work. Then it pisses people off (P1)”.*

##### Design and methods

Participants believe that quantitative methods alone are not sufficient to evaluate costs and benefits of OHM: “*There are many effects of OHM that cannot be quantified. And there are many influences that cannot be included or taken into account. That is why a focus on a quantitative evaluation is not expedient (P2)*”. Single, rare events such as a change in leadership have large impact on outcomes (e.g. engagement, absenteeism). From the participants’ point of view, this cannot be controlled by (cluster) randomised trials: *“[…] this typical intervention and control group are simply impossible in this type of intervention […]. Because it is also ethically unacceptable. […] apart from the fact that there are never two identical teams in the same place at the same time (P8)”.* The processes, influences from the environment (see Sect. 4), as well as intangible outcomes must be qualitatively evaluated and taken into account in the final EE. Classical methods (reductionism) reach their limits in OHM because it is about complex, social systems (i.e. systems thinking).

##### Perspective

Regarding the analytical perspective, there were different opinions. Statements such as: *“we benefit not only as a company, we benefit as a society (P13)”* or *“[…] the potential damage is transferred to the society while the benefits remain within the company (P8)”* speak for a societal perspective (i.e. considering all costs, regardless who covers them). Other participants think that because the company bears the costs, this should also be the focus of the analysis: *“I do think that you should take the perspective that is right for the decision maker (P6)”.*

##### Modelling approaches

Larger companies in particular, work with models and scenarios: *“[…] We made scenarios based on demographic trends. And we said, what if we do nothing at all? (P2)”.* Company-specific figures are combined with information from the literature to estimate the potential of an intervention: *“You measure what is possible in practice, and then you also take the literature where certain relationships are proven (P1)”.* The participants are aware of the uncertainty of such scenarios: *“[…] of course, this is always subject to very large uncertainties. It’s about getting a feel for it. And it’s not about the exact number. But to see through scenarios like that, what could it cost and what could it do (P6)”.*

##### Time horizon

“*OHM happens slowly*” was the second most used code. Health and especially corporate success can only be evaluated in the long-term, over several years: *“It’s such a long story with this OHM. It’s a slow work. This is not something that happens quickly (P13)”.* This inertia of OHM is perceived as inhibiting: *“This is always difficult, though. Because I have the feeling that people always expect a short-term benefit (P5)”.*

The optimisation of demands and resources is mentioned as a medium-term outcome: *“This resources-demands-ratio. We always discussed: is it proximate? Is it intermediate? It is rather intermediate (P3)”.*

##### Population

In a majorly healthy population, the effect potential is limited: *“These are always healthy target groups. And there was always the discussion to show the return-on-investment in people who are healthy. You know that you can show that they remained healthy (P3)”* or *“[…] absences will not always go down (P8)”.*

People with the greatest potential for impact are often those, who do not implement the measures: *“Mostly it is like this, people who already move more, they also participate more (in a physical activity program) (P7)”.*

##### Impact models

Participants use impact models to establish specific pathways through which OHM-interventions influence health and company success: *“[…] you have to show these chains of effects. Our impact model has different levels. So, you start somewhere, then it has an influence on demands and resources. Then you can measure if something has changed. That is like the first level. And then comes the second level, how healthy the people are. And only at the end comes the outcome at the company level (P2)”*. Impact models are also used for diagnostic reasons: *“So I work with the model backwards. I look at the health indicators, perhaps the absences, resources, and stress. And then I think about where we can start (P1)”.* These models help to better understand the overall dynamics of these complex systems. Environmental factors can also be considered. The companies have either developed their own impact model or they adapt the model provided by HPS.

The majority of the participants believe that EEs of individual, isolated interventions are difficult because different interventions interact with each other and have a multidimensional effect (i.e. effect on several outcomes): *“[…] because in practice, you often do several things. […] to evaluate an impact chain of only one intervention, that is not possible from my point of view. From my point of view, it is never possible to evaluate interventions individually. Because you simply have too many influencing factors (P1)“. “So, you then simply have the overall effect at the end (P7)”.*

## Discussion

In this qualitative study we discussed the currently used methods of EEs in the field of OHM with 13 OHM-specialists. Furthermore, their opinions on the economic dimensions of OHM were investigated. The aim was to collect ideas to improve quality and validity of future EEs in this area. None of the participants had specific experiences with EEs. However, they provided surprisingly detailed inputs on the topic, indicating an appropriate selection of participants and suggesting that our research question is relevant and of interest in the practical field.

Understanding of OHM was one of the five main themes which emerged from the data. The respondents emphasised that OHP is essential but not sufficient for effective OHM. In fact, the embedding of interventions in the existing structures of the organisation and in particular the development of a “culture of health” is increasingly emphasised in the literature [30, 31]. For example, Kent et al. [30] aimed to identify key success elements of employer-sponsored OHM programs and they concluded that more efforts are needed to build cultures of health and excellent communication strategies in workplace settings. However, quantifying these structural costs (Fig. 1, marking 2) to consider them in EEs is a major challenge.

For participants, health should be the primary reason for undertaking OHM, not the monetary benefits. Interestingly, guidelines on EEs of occupational safety and health interventions explicitly point out that too many EEs focus exclusively on financial outcomes without the consideration of health [32]. Participants argued that healthy employees must have a positive impact on the company in the long term. Indeed, studies confirmed the positive relationship between happy, healthy workers and company performance [33–35]. However, as these studies followed correlational study designs, they are criticised for lacking causality. Vice versa, for example, there is also evidence that productivity growth may have detrimental effects on health as a typical approach to cut costs is downsizing (fewer people do the same work), which in turn has been shown to result in higher job-demands and higher presenteeism [36, 37]. 

However, the OHM-specialists also argued that health improvements (Fig. 1, marking 5) are sometimes hard to measure, especially in a predominantly healthy population and in the short term. A systematic review supports this statement as EEs considering health-related outcomes were less likely to be cost-effective or cost beneficial compared to EEs considering productivity-based outcomes [11]. According to the participants, future EEs could therefore focus more on job-resources and job-demands. For example, the recently published resources-demands ratio [38] may be used for a CEA.

On the one hand, there is strong evidence regarding the relationships between demands, resources, and health [39]; on the other hand, effects can be expected in the medium term which makes studies much more feasible.

From the interviews it became clear that productivity is the most important economic dimension in the OHM-context. However, participants see difficulties in quantifying productivity. In fact, a systematic review confirmed that the methods for measuring and valuing absenteeism and presenteeism in EEs remain controversial [12]. Furthermore, participants perceived that intangible benefits represent the larger part of the effect (Fig. 1, marking 8) and should therefore not be underestimated. One could argue that there is bias in this regard because respondents want to avoid numerical quantification of OHM programs, as that would provide a management tool for the evaluation of their own performances. Nonetheless, a study which investigated methodological challenges of EEs in the field of OHM also emphasised the importance of intangible benefits [40]. Furthermore, five of the participating companies mentioned their OHM strategy in their annual business report. In fact, this was done in all five reports explicitly to enhance their image (which was considered as intangible benefit by the OHM-specialists).

There were only 9 codes related to the analytical perspective. Still, the participants were aware that there can be transfers of costs and benefits. Thus, it may be that one stakeholder bears the costs and another enjoys the benefits (and vice versa). Taking multiple perspectives into account is therefore important for future EEs because in certain situations it can legitimise an intervention being cross-subsidised, e.g. by the public sector, because otherwise it would not be implemented.

Evaluation of OHM was another main theme. The experts, of which 11 had at least a Master’s degree, expressed doubts regarding the scientific methods of EEs. Randomised controlled trials (RCT) are considered the gold standard to evaluate effectiveness of interventions and EEs alongside RCTs have been performed in the field of OHM [41, 42]. Nevertheless, EEs alongside RCTs that evaluate OHM interventions have been questioned by participants because (a) in reality, there is rarely only one intervention implemented, (b) multiple interventions interact with each other, (c) interventions may have multidimensional effects, (d) clusters may not be comparable, and (e) RCTs normally have a too limited time horizon to capture relevant effects. However, according to the participants, the greatest challenge for the evaluation of OHM are the constant environmental influences from the side of the company as well as from the side of the employees (Fig. 1, markings 9 and 10). Indeed, it is increasingly questioned whether RCTs are appropriate in the field of OHM [32, 43]. More recent studies acknowledg e that the relationship between work, environmental factors, health, and company success is complex, and that this complexity might be ignored or artificially reduced in RCTs [43, 44]. Guidelines on the evaluation of complex interventions increasingly point to the limits of pure reductionism and emphasise the potential of system thinking [45].

While acquiring the FWS-label, all participants dealt with the same criteria established by HPS. Furthermore, at the time of the interviews, almost all of them were working with an impact model as proposed by HPS. This common ground may explain why participants agreed on many discussion points and why saturation was already reached after 12 interviews. This selective choice limits the generalisability to companies without the FWS-label and to companies outside Switzerland. However, generalisability was not the main aim of this qualitative study, but rather to gain in-depth data from OHM-specialists.

Beside OHM-specialists, future research should also consider views and opinions of other stakeholders of OHM. This may allow to achieve a full picture of critical parameters and levels of uncertainty and thus, to make recommendations for future EEs.

## Conclusions

Meaningfulness and purpose of OHM are more important amongst the questioned OHM-specialists than facts and figures from EEs. Costs and benefits of OHM were perceived significantly different than how they are represented in current EEs. According to the participants, most benefits cannot be quantified und thus, monetised. These intangible benefits as well as critical influencing factors during the process should be assessed qualitatively and considered in EEs when using them as a legitimation basis vis-à-vis decision makers.

## Supplementary Information

**Additional file 1.**

## Data Availability

The datasets generated and/or analysed during the current study are not publicly available for data protection reasons. Data is available from the corresponding author on reasonable request.

## Abbreviations

GDP: Gross domestic products
EE: Economic evaluation
OHM: Occupational health management
CBA: Cost-benefit analysis
CEA: Cost-effectiveness analysis
ROI: Return-on-investment
ICER: Incremental cost-effectiveness ratio
HPS: Health Promotion Switzerland
FWS: Friendly Workspace (label)
RCT: Randomized controlled trial

## Declarations

The study was approved by the local ethics committee (Ethics committee Bern, Switzerland, BASEC-Nr: Req-2020-01115). All participants provided written informed consent.
