# Supplementary material for: What are the economic dimensions of occupational health and how should they be measured? A qualitative study
Source: BMC Public Health. 2022 Jul 15;22:1362. doi: 10.1186/s12889-022-13659-y (PMC9284955; doi:10.1186/s12889-022-13659-y)
Supplement: Supplementary file 1 — Additional file 1. [file 12889_2022_13659_MOESM1_ESM.docx]

# Additional file 1

1. **Question guide**

**Introduction**

There are scientific studies which aim to prove the benefits of occupational health management (OHM). Furthermore, economic evaluations of OHM-interventions aim to determine their cost-benefit ratio. We conducted two literature reviews, to summarise the methods which were used to analyse the costs and benefits of WHP-interventions. We found that there are two main types of economic evaluations: Cost-effectiveness analyses considering the costs and the effects of at least two alternatives (i.e. OHM versus no OHM). Effectiveness is measured in natural units (e.g. stress level on a scale from 0 to 100). If the intervention is more effective, one calculates the incremental costs (e.g. the additional costs per one-point stress reduction).

Cost-benefit analyses also measure costs and effects of at least two alternatives, but they express the effects in monetary units. For example, absenteeism statistics are used to determine the loss of working time, which is then multiplied with a monetary value (e.g. hourly or daily wage). Consequently, costs and benefits are presented in monetary terms which allows the calculation of typical metrics such as the net-benefit or the return-on-investment.

In almost 60 studies which we analysed and compared with each other, we noticed the following points in particular:

- The definition of “relevant” costs and benefits depends on the perspective from which the economic analysis is performed. OHM-interventions may be analysed from the employer’s, the employees’ or the societal perspective.
- Even studies which performed the economic evaluation from the same perspective, used different definitions of costs and benefits. For example, about half of the studies considered productivity loss as the amount of absenteeism and the other half of studies as the sum of absenteeism and presenteeism. However, there are also studies which do not even take productivity losses into account.
- From the employer’s perspective, solely improved productivity (less absenteeism and presenteeism) were considered as benefits.

We believe that the economic dimensions of OHM go beyond those that are taken into account in current studies. For this reason, we would like to hear your meanings and views on the methods described above in the following interview. The main goal of the interview is to learn more about the economic dimension of OHM from your perspective and your experience in this field.

The interview will last about 60 minutes and you may ask questions at any time if there are uncertainties.

| **Question type** | **Question** | **Context** |
| --- | --- | --- |
| Opening | How did you get in contact with OHM and what are your current tasks in this field? | Icebreaker |
| Intro | Why do you think your company engages in OHM? | General conviction |
|  | Which OHM-strategies are you implementing and how are they perceived by the employees? | Perception employees |
|  | In your opinion, how are the OHM-interventions perceived by the employer or the directors?   - How do you experience the management’s commitment to OHM? | Perception employer |
| Key | In what way do you think that OHM-interventions influence health and motivation of the employees?   - How is health of the employees measured? - In your opinion, are there any other benefits for employees? | Benefits employees |
|  | In what way do you think the (successful) OHM-interventions pay off for the company?   - What elements are the benefits for the company made up of? - How are the benefits for the company measured? | Benefits employer |
|  | In your opinion, are there any aspects of OHM, which affect employees negatively? | Costs employees |
|  | In your opinion, are there any aspects of OHM, which affect the employer or the company negatively?   - How does the company handle the costs of WHP? - Are there OHM-interventions, which are not put into practice due to cost reasons? | Costs employer |
|  | In most economic evaluations, absenteeism costs are calculated by multiplying the number of lost work hours by the hourly wage. What are your thoughts regarding this procedure? | Absenteeism |
|  | Imagine you had to conduct an economic evaluation of the OHM-strategy in your company.   - What elements would the costs of these interventions be made of? - Which factors at the benefit level would you consider to be useful in an economic evaluation? - What opportunities do you see in carrying out economic evaluations of OHM? And where are the difficulties for you? - It is not always possible to carry out economic evaluations. What alternatives do you see for the evaluation of OHM? | Summary |
| Ending | Do you have any further questions or remarks? |  |

OHM: occupational health management
